# All-in-one nerve guidance: transplanting a gradient scaffold with immobilized Schwann cells for peripheral nerve regeneration

**DOI:** 10.1016/j.mtbio.2026.103294

**Published:** 2026-05-28

**Authors:** Shengwen Zhu, Rui Cui, Shuai Qiu, Xi Zhang, Jingxin Ma, Wan Duan, Peiyao Li, Daping Quan, Zehong Yang, Sien Zhang, Zilong Rao, Ying Bai

**Affiliations:** a Guangdong Engineering Technology Research Centre for Functional Biomaterials, Key Laboratory for Polymeric Composite & Functional Materials of Ministry of Education, School of Materials Science and Engineering, Sun Yat-sen University, Guangzhou, 510006, China; b Department of Orthopedics, The Eighth Affiliated Hospital, Sun Yat-sen University, Shenzhen, 518033, China; c Hospital of Stomatology, Guanghua School of Stomatology, Sun Yat-sen University, Guangdong Provincial Key Laboratory of Stomatology, Guangzhou, 510055, China; d Department of Radiology, Sun Yat-Sen Memorial Hospital, Sun Yat-Sen University, Guangzhou, 510120, China

**Keywords:** Peripheral nerve regeneration, Schwann cell, Decellularized extracellular matrix, Neurotrophic factors, Microgels

## Abstract

Peripheral nerve injury (PNI), especially those with long-distance transected defects, remains a major clinical challenge due to limited regenerative capacity associated with inadequate endogenous Schwann cell (SC) support. Here, we developed a biomimetic nerve graft with a defined spatial gradient of immobilized SCs to facilitate effective transplantation and axonal guidance, thus enhancing peripheral nerve regeneration. SCs were initially encapsulated within decellularized nerve matrix (DNM) microgels using a customized flow-focusing microfluidic device. The DNM microgels supported good viability, facilitated cellular proliferation, and preserved the repair phenotype of the encapsulated SCs, while maintaining their advantageous paracrine activity conducive to axon extension. Furthermore, these microgels were incorporated into the density-gradient pores of a pre-designed scaffold, resulting in a gradient SC-laden scaffold, designated as MGs@G-scaffold. Notably, the spatially graded SCs within the MGs@G-scaffold created a stable environmental gradient of neurotrophic factors, thereby providing sustained biochemical cues to guide axonal elongation. Finally, when transplanted into a 15-mm rat sciatic nerve defect model, the SC-laden scaffolds exhibited significantly improved axonal regeneration, remyelination, and functional recovery compared with the SC-free scaffolds. Additionally, MGs@G-scaffolds outperformed scaffolds with uniform SC distribution (MGs@H-scaffolds), achieving therapeutic outcomes comparable to autografts. Overall, this study demonstrates that transplanting spatially graded SCs embedded in a tissue-engineered bioactive scaffold offers sustained ongoing support for cells, gradient biochemical signals, and a pro-regenerative microenvironment for nerve regeneration and functional recovery, which holds great promise for long-distance PNI repair.

## Highlights

• Schwann cell transplantation and growth factor gradient were combined in an engineered scaffold.
• Decellularized nerve matrix microgels maintain the repair phenotype of transplanting Schwann cells.
• Sustained, graded release of neurotrophic factors was achieved using immobilized Schwann cell-laden microgels.
• The gradient scaffold promotes axonal extension, remyelination, and functional recovery after peripheral nerve injury.

## Graphical abstract

**Figure:**
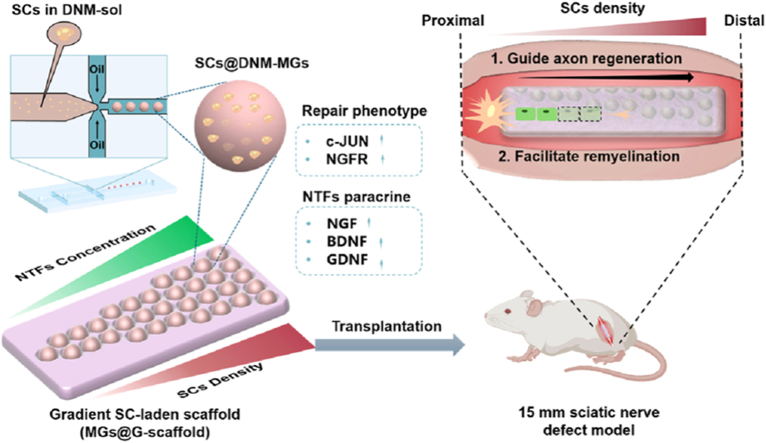

## 1 Introduction

Peripheral nerve injury (PNI) occurs due to accidents, fractures, and non-traumatic causes like genetic disorders, drug toxicity, and immune responses, affecting millions worldwide annually [1]. Small nerve gaps (under 8 mm) are often repaired with direct end-to-end anastomosis, while larger gaps generally require implantable grafts to bridge the nerve ends and promote regeneration. Autologous nerve grafting is regarded as the clinical “gold standard,” but its use is limited by donor site morbidity, structural mismatch, and suboptimal functional recovery, especially in long nerve gaps [2]. Chronic and long-segment injuries often lead to the loss of sustained neurotrophic factors and disruption of native basal lamina tubes, creating a hostile microenvironment that lacks both biochemical and physical guidance. These deficiencies substantially limit axonal sprouting and result in mismatched reinnervation between nerve stumps, thereby impacting functional outcomes [3]. Consequently, the development of engineered nerve grafts capable of reconstructing a supportive, pro-regenerative microenvironment has emerged as a primary objective in peripheral nerve tissue engineering.

Following PNI, Schwann cells (SCs) play essential roles in multiple regenerative processes, including clearance of myelin debris, formation of Büngner bands to guide axonal regrowth, and secretion of neurotrophic factors (NTFs), including nerve growth factor (NGF), brain-derived neurotrophic factor (BDNF), and glial cell line-derived neurotrophic factor (GDNF) [[4], [5], [6], [7]]. To maximize these regenerative functions, mature myelinating SCs dedifferentiate into a proliferative repair phenotype after injury. This phenotypic transition is orchestrated by rapid upregulation of the transcription factor c-JUN, a master regulator that triggers the repair-associated genetic cascades [[8], [9], [10]]. Specifically, c-JUN promotes the expression of regeneration-associated markers, including NGFR (p75NTR) and neurotrophic factors, while suppressing myelination-related genes, including PMP22, MAG, and MPZ, thereby maintaining SCs in a regenerative state. As a result of this transcriptional reprogramming, repair-type SCs demonstrate increased proliferative and migratory abilities, along with substantial paracrine secretion of NTFs. These combined effects significantly contribute to the formation of Büngner bands, axonal regeneration, and subsequent myelination [11]. However, numerous SCs experience dysfunction, apoptosis, or even death following PNI. Consequently, the remaining endogenous SCs offer insufficient support for nerve regeneration, especially in cases of long-segment PNI. Therefore, transplanting exogenous SCs, especially repair-type SCs, can be an effective approach to achieving functional recovery. However, the hostile inhibitory microenvironment, including immune responses and oxidative stress, often impairs the survival and function of transplanted SCs, especially their capacity to sustain the repair phenotype [12].

Biomaterial-based cellular transplantation strategies have emerged as promising solutions by enabling targeted cell delivery, providing essential biological signals to promote cell viability, and modulating the surrounding microenvironment to support regeneration [13,14]. Naturally derived hydrogels, including collagen, hyaluronic acid (HA), gelatin methacryloyl (GelMA), fibrin, and decellularized extracellular matrix hydrogels (dECM-gels), have demonstrated capabilities in regenerative medicine [[15], [16], [17]]. Among these, the dECM hydrogels, which retain most of the critical tissue-specific ECM components, have attracted significant attention for their resemblance to native tissues. Specifically, a dECM-gel derived from porcine sciatic nerves (decellularized nerve matrix hydrogel, DNM-gel) has shown remarkable ability to support SC proliferation, migration, and remyelination *in vitro*, and to promote axonal regeneration *in vivo*, as demonstrated in our previous research [[18], [19], [20]]. Presumably, the DNM-gel, by providing a regenerative microenvironment, can help maintain the repair phenotype of transplanted SCs, thereby enhancing their contribution to axonal regeneration.

In addition to the physical support provided by Büngner bands and the potential for myelination, transplanted SCs might also deliver spatially varying biochemical cues, including the secretion of NTFs. Emerging evidence indicates that, beyond merely the presence of neurotrophic factors, their concentration gradients can significantly influence axon extension and trajectory. Growth cones can sense these guidance-molecule gradients and tend to extend preferentially toward regions of higher factor concentrations [21]. To combine these instructive cues, various fabrication strategies have been developed to incorporate spatially controlled growth factor gradients within nerve grafts, including electrospinning, 3D printing, and post-fabrication gradient infusion techniques [[22], [23], [24]]. Although these methods enable the initial establishment of biochemical gradients, direct immobilization of growth factors often results in transient, non-renewable signaling due to rapid diffusion and degradation following implantation [25]. Such short-lived gradients are inadequate for long-segment PNI, which requires sustained guidance to support extended SC migration and axonal regeneration. Notably, repair-phenotype SCs act as natural reservoirs of neurotrophic factors, continuously secreting them paracrinally. Consequently, designing a nerve graft with a gradually changing spatial arrangement of SCs could facilitate the development of a self-maintaining neurotrophic gradient, offering ongoing biochemical guidance for PNI repair. However, constructing an engineered gradient scaffold with a specific spatial distribution of SCs remains a significant challenge.

Most biomaterial carriers are recognized for their ability to encapsulate living cells and precisely place them within specific regions, given the fragility and limited manipulability of these cells [26]. In previous work, we described a series of microscale dECM gels derived from the nervous system that demonstrated strong capabilities for cell encapsulation, transplantation, and bioprinting while maintaining sustained cellular viability and function [[27], [28], [29]]. Specifically, the SC-laden DNM microgels can be effectively incorporated into GelMA bioinks to achieve high post-printing cell viability and modular co-culture systems with controllable cellular distribution [28]. Furthermore, we demonstrated that neural stem/progenitor cells encapsulated in the decellularized spinal cord matrix microgel can be 3D cultured into a pre-mature state prior to transplantation, thereby enhancing their survival and neuronal maturation at the lesion sites following spinal cord injury [29]. These findings collectively indicate that dECM-derived microgels offer distinct benefits as cytoprotective and bioactive carriers for precision cell-based graft engineering.

In this study, primary rat SCs were encapsulated in DNM microgels (SCs@DNM-MGs) using a custom flow-focusing microfluidic system and pre-incubated to maintain their repair phenotype. These cultured SCs@DNM-MGs were then embedded into a composite scaffold with a specific density gradient (MGs@G-scaffold), allowing a gradual spatial distribution of repair-phenotype SCs along the proximal-distal axis. *In vitro* analyses were conducted to verify the preservation of phenotypic characteristics of encapsulated SCs and to characterize the neurotrophic factor gradients produced from the MGs@G-scaffold. Additionally, the scaffolds were co-cultured with dorsal root ganglia (DRGs) to assess the ability of the cell-mediated gradients to facilitate directed axonal outgrowth. Finally, the MGs@G-scaffolds were incorporated into biodegradable conduits and subsequently implanted into a 15-mm sciatic nerve defect model to evaluate regenerative efficacy and functional recovery *in vivo*.

## 2 Experimental section

### 2.1 Ethics

All animal procedures in this study were approved by the Laboratory Animal Center of Sun Yat-sen University (Approval no. SYSU-IACUC-2025-001795) and conducted in accordance with the *Laboratory Animal Guidelines for Ethical Review of Animal Welfare* (GB/T 35892-2018).

### 2.2 SCs/GFP-SCs isolation and culture

Primary SCs and green fluorescent protein-labeled SCs (GFP-SCs) were isolated from wild-type Sprague-Dawley rats (Laboratory Animal Center of Sun Yat-sen University, Guangzhou, China) and GFP-transgenic Sprague-Dawley rats (Beijing Vitalstar Biotechnology, Beijing, China), respectively. Sciatic nerves were excised and immediately transferred to ice-cold DMEM medium (Gibco, USA) containing 1% penicillin-streptomycin. After removing connective tissues, nerve fragments were digested with 0.05% collagenase I (Sigma-Aldrich, USA) for 60 min at 37 °C, followed by 0.25% trypsin for 30 min. The cell suspension was seeded onto culture flasks for 20 min to allow fibroblasts to adhere. Non-adherent SCs were collected and cultured in DMEM/F12 medium supplemented with 10% fetal bovine serum (FBS; Corning, USA).

### 2.3 Preparation of DNM

Fresh sciatic nerves were harvested from miniature pigs and decellularized using 3.0% Triton X-100 and 4.0% sodium deoxycholate, then rinsed with sterile water. Lipids were extracted with a mixture of ethanol and dichloromethane (v/v ∼1:2). The tissues were lyophilized and ground into powder using a Thomas-Wiley Mini-Mill (Thomas Scientific, USA). The resulting DNM powder was digested in 0.01 M HCl containing pepsin (Sigma-Aldrich, USA) for 4 h and then sterilized by lyophilization. After redissolution in 0.01 M HCl and adjustment of the pH to 7.4, 10× DMEM solution was added to obtain the DNM precursor solution (DNM-sol), which was stored at 4 °C until use.

### 2.4 Preparation of SCs@DNM-MGs

The SCs loaded DNM microgels (SCs@DNM-MGs) were fabricated using a previously described multi-stage temperature-controlling microfluidic system (MSTC-MS) with minor modifications [27]. All equipment was sterilized by autoclaving before use. After two days of culture, primary SCs were dissociated into single cells using 0.25% trypsin (Sigma-Aldrich, USA) and mixed with DNM-sol (7.5 mg mL^−1^) at a cell density of 1.5 × 10^7^ mL^−1^ to obtain the water phase. HFE 7500 oil (3M, USA) containing 0.5% (w/v) PEG-Krytox-PEG surfactant (RAN Biotech, USA) was used as the oil phase. Both phases were loaded into syringes mounted on a multi-channel injection pump (WH-SSP, Wenhao Co., China) and injected at flow rates of 0.35 mL h^−1^ (water phase) and 15 mL h^−1^ (oil phase), respectively. In particular, the water-phase syringe and microfluidic chip were placed in a portable refrigerator to maintain the temperature at 6 °C and prevent DNM-sol gelation. The water phase was sheared into droplets and then passed through a silicone tube immersed in a 37 °C water bath to induce gelation, yielding SCs@DNM-MGs. The emulsion was collected in centrifuge tubes, and the oil phase at the bottom was removed. A 20% (v/v) solution of 1H,1H,2H,2H-perfluoro-1-octanol (PFO, Aladdin, China) in HFE 7500 was added to the tube to demulsify. The SCs@DNM-MGs were gently resuspended in DMEM/F12 medium supplemented with 10% FBS, transferred to ultra-low attachment six-well plates (Corning, USA), and cultured at 37 °C in a humidified incubator with 5% CO_2_. The medium was replaced every 24 h. Morphology and the number of encapsulated SCs in the DNM-MGs were observed and counted under an optical microscope (TS2-LS, Nikon, Japan) after preparation.

To 3D culture the SCs in bulk DNM-gel (denoted as SC@DNM-gel), primary SC spheroids were dissociated into single cells and embedded in DNM-sol at a density of 1.5 × 10^7^ mL^−1^. The mixture was transferred to six-well tissue culture plates (TCP, Corning, USA) and incubated at 37 °C for 10 min to induce gelation. The SCs were then incubated at 37 °C in a humidified incubator with 5% CO_2_, with the medium replaced every 24 h.

### 2.5 Cell viability and proliferation assays

The viability of SCs cultured in bulk DNM-gel, DNM-MGs, and TCP was assessed using the Live/Dead assay (Meilunbio, China) on days 1 and 3, respectively. Briefly, after removing the culture medium, samples were washed three times with PBS and incubated with Calcein-AM (1:2000, for live cells) and propidium iodide (1:2000, for dead cells) at room temperature for 20 min. The stained samples were visualized using a laser-scanning confocal microscope (LSM710 NLO, ZEISS, Germany), and cell viability was calculated as the ratio of live cells to the total number of cells.

Cell proliferation was evaluated using EdU staining after 5 days of culture with a Cell-Light EdU Apollo488 In Vitro Kit (Ribo Biotechnology, China), following the manufacturer's instructions. EdU solution was added to the culture medium at a dilution of 1:2000 and incubated for 24 h. SCs cultured in DNM-MGs, bulk DNM-gels, and TCP were fixed with 4% paraformaldehyde and blocked with 5% (w/v) bovine serum albumin (BSA) and 0.5% Triton X-100. After washing three times with PBS, samples were incubated with the EdU staining working solution for 30 min at room temperature. Cell nuclei were then stained with DAPI (Servicebio, China).

### 2.6 Immunofluorescence staining

SCs were fixed, permeabilized, and blocked as described above, then incubated overnight at 4 °C with the following primary antibodies: mouse anti-S100 (1:200; Immunoway, China) for SCs identification, anti-c-JUN (1:200; Immunoway, China), and anti-tumor necrosis factor receptor superfamily member 16 (NGFR, 1:200; Immunoway, China) for SCs repair phenotype identification. After washing three times with PBS, samples were incubated with anti-rabbit Alexa Fluor 488 and anti-mouse Alexa Fluor 594 (Thermo Fisher Scientific, USA) for 1 h at room temperature. Nuclei were counterstained with DAPI and visualized using a fluorescence microscope (TS2-LS, Nikon, Japan).

### 2.7 Western blot

SCs were lysed in RIPA buffer (Meilunbio, China) supplemented with a protease inhibitor cocktail (Meilunbio, China). After incubation in a metal bath at 95 °C for 10 min, SDS-PAGE was performed on a 10% polyacrylamide gel (Epizyme Biotech, China) at 120 V for 1 h. The proteins were then transferred onto PVDF membranes at 400 mA for 30 min. Membranes were blocked with a rapid blocking solution for 30 min and incubated with antibodies against c-JUN (Immunoway, China), Vinculin (Immunoway, China) overnight at 4 °C. After washing, membranes were incubated with HRP-conjugated secondary antibodies for 1 h at room temperature. Protein bands were detected using an Amersham ECL Select Western Blotting Detection Reagent (Biosharp, China). Band intensities were quantified with ImageJ software (NIH, USA) and normalized to vinculin.

### 2.8 Quantitative real-time polymerase chain reaction (qRT-PCR)

Total RNA was extracted from SC spheroids using a MiniBEST Universal RNA Extraction Kit (Takara, Japan) according to the manufacturer's instructions and reverse-transcribed into cDNA with Primescript Master Mix (Takara, Japan). Quantitative real-time PCR was carried out using TB Green Premix ExTaq II (Takara, Japan) on a QuantStudio™ 5 System (Thermo Fisher Scientific). The following primers were used: c-JUN, forward, 5′- CCAACCAACGTGAGTGCAAG-3′ and reverse, 5′- GAGGGCATCGTCGTAGAAGG-3′; NGF-β, forward, 5′- GAGCGCATCGCTCTCCTT-3′ and reverse, 5′-CTGTGTACGGTTCTGCCTGT-3′; GDNF, 5′-TGTTCTCCTCTCCTGGCTGT-3′ and reverse, 5′-CTTCCTCCTCGAGTGTCGTG-3′; BDNF, forward, 5′-CTTGGAGAAGGAAACCGCCT-3′ and reverse, 5′- GTCCACACAAAGCTCTCGGA-3′. β-actin was used as the internal reference gene.

### 2.9 Preparation of GelMA/DNM-P composite hydrogel

Gelatin methacryloyl (GelMA) was synthesized as previously described [28]. Briefly, 10 g of gelatin (Type A, 300 bloom, Sigma-Aldrich, USA) was dissolved in 100 mL of PBS at 60 °C to obtain a 10% (w/v) solution. Then, 10 mL of methacrylic anhydride (Sigma-Aldrich) was added dropwise at about 0.5 mL/min while stirring at 50 °C. After reacting in the dark for 3 h, the mixture was diluted with 5× PBS and dialyzed against deionized water at 37 °C for 5 days, with the water replaced every 12 h. The final product was lyophilized and stored at −20 °C until use.

GelMA/DNM-P composite hydrogel was prepared by dispersing DNM powder (DNM-P) into GelMA solution. Briefly, 500 mg of GelMA was dissolved in 10 mL of PBS containing 0.3% (w/v) lithium acylphosphonate (LAP, Macklin, China), then 500 mg of DNM powder was added to the solution, resulting in final concentrations of 5% (w/v) GelMA and 5% (w/v) DNM-P. The mixture was then homogenized by vortexing for 1 min and sonicated for 5 min until uniform. The composite hydrogel was stored at −40 °C and rewarmed to 25 °C before use.

### 2.10 Preparation of MGs@G-scaffold and MGs@H-scaffold

GelMA/DNM-P scaffolds with either an axially gradient or uniform pore distribution were fabricated using customized polydimethylsiloxane (PDMS) templates. Briefly, 100 μL of GelMA/DNM-P composite hydrogel was added to a PDMS mold with a patterned micropillar array matching the pore structure of the respective scaffolds. The hydrogel was then covered with a PDMS substrate and gently pressed to remove any excess hydrogel. The composite hydrogel was photocrosslinked using UV light (365 nm, 5 mW/cm^2^) for 15 min, then the scaffold was peeled off from the mold. To assemble SC@DNM-MGs within the scaffold pores, GelMA/DNM-P scaffolds were placed flat in PDMS wells (15 mm in length, 6 mm in width, 5 mm in height) and immobilized by adding DMEM/F12 containing 0.01% DNM-sol. After incubation at 37 °C for 1 h, the medium was removed, and SC@DNM-MGs suspended in DMEM/F12 containing 0.01% DNM-sol were added to the wells. The devices were then incubated at 37 °C for an additional 2 h to enable complete deposition of SC@DNM-MG into each pore. The GelMA/DNM-P scaffolds with either a gradient distribution (termed MGs@G-scaffold) or a uniform distribution of SC@DNM-MG (termed MGs@H-scaffold) were subsequently peeled off from the wells and rinsed with SC culture medium.

### 2.11 Enzyme-linked immunosorbent assay (ELISA)

To assess growth factor secretion by SCs, culture supernatants from SCs grown on TCP and bulk DNM-gel were collected after 5 days. The concentrations of NGF, BDNF, and GDNF were measured using ELISA kits (Fine Biotech, China), and absorbance was read at 450 nm with a microplate reader (Multiskan Mk3, Thermo Fisher Scientific, USA).

To evaluate the gradient distribution of growth factors secreted from SC@DNM-MGs, MGs@G-scaffolds were cut into three segments along the gradient of decreasing SC density, while MGs@H-scaffolds were sectioned into three corresponding regions. Each segment was incubated in 500 μL of SC culture medium at 37 °C for 7 days. Culture supernatants were collected on days 3, 5, and 7, with fresh medium replaced every 24 h. Growth factor concentrations were measured by ELISA as described above.

### 2.12 Dorsal root Ganglion (DRG) isolation and Co-culture

DRGs were isolated from newborn Sprague Dawley (SD) rats, and the residual nerve roots were cut off under a stereomicroscope. To verify the growth factor gradients secreted from the MGs@G-scaffold, DRGs were first seeded into 24-well culture plates and cultured in DRG medium (neurobasal medium supplemented with 2% B27, 1% L-glutamine, and 1% penicillin-streptomycin). After DRG adhesion, the culture medium was replaced with conditioned medium supplemented with supernatants from the three segments of both MGs@G-scaffold and MGs@H-scaffold. To assess how growth factor gradients influence axonal outgrowth, a customized PDMS co-culture device was used to immobilize the MGs@G-scaffold and MGs@H-scaffold. The DRGs were seeded near the short edge, roughly at the bottom of the device, to mimic an *in vivo* situation where axons grow from proximal to distal at the injury site. The co-culture devices were filled with DRG culture medium, which was refreshed daily, and maintained for at least 14 days.

DRGs were also cultured in the conditioned medium supplemented with day 7 culture supernatants from the three respective segments from MGs@G-scaffold as an additional assessment of the gradient distribution of growth factors secreted by SC@DNM-MGs. After 14 days of culture, DRGs were fixed with 4% paraformaldehyde in PBS for 20 min. Samples were rinsed with PBS, permeabilized, and blocked with 0.5% Triton X-100 and 5% BSA for 30 min. DRGs were then incubated with primary antibodies against NF200 (1:150) overnight at 4 °C. After rinsing with PBS, samples were incubated with secondary antibodies conjugated to Alexa Fluor 488 (1:1000) for 1 h at room temperature, followed by nuclear counterstaining with DAPI (1:2500) for 20 min. Fluorescence images were acquired with a laser-scanning confocal microscope (Zeiss, Germany).

The maximum axon outgrowth length was measured using ImageJ software. The axon with the farthest extension was first identified, and a straight line was drawn from the center of the DRG spheroid to the tip of the selected axon. The intersection point between this line and the outer boundary of the DRG spheroid was then determined. The distance from the axon tip to this intersection point was defined and recorded as the maximum axon outgrowth length.

### 2.13 Fabrication of nerve guidance conduits

Before performing the animal experiments, non-cytotoxic electrospun poly(L-lactic acid)-co-poly(trimethylene carbonate) (PLLA-co-PTMC) nanofibrous tubes were prepared as protective conduits to bridge nerve gaps and prevent damage, such as bending, tearing, or rupture, caused by surrounding tissues [30,31]. After sterilization with Co^60^ irradiation, homogeneous GelMA/DNM-P scaffolds without SC@DNM-MGs deposition (H-scaffold), MGs@H-scaffolds, and MGs@G-scaffolds were wrapped longitudinally around 20 G syringe needles. The needle-scaffold assemblies were then inserted into PLLA-co-PTMC protective tubes, after which the needles were carefully withdrawn using tweezers to prevent scaffold displacement. The resulting nerve conduits were stored in SC culture medium before transplantation.

### 2.14 Scanning electron microscopy (SEM)

For SEM characterization, scaffolds and horizontal semi-thin slices of nerve conduits were lyophilized and mounted on the sample stage using conductive tape. After sputter coating with platinum, the morphology of SC@DNM-MGs within the GelMA/DNM-P scaffolds was observed with a scanning electron microscope (HITACHI S-4800, Tokyo, Japan) operated at an accelerating voltage of 10 kV.

### 2.15 Animals

A total of 56 Sprague Dawley (SD) rats (2 months old, weighing 200-250 g) were obtained from the Animal Center Laboratory of Sun Yat-sen University (Guangzhou, China). The rats were housed and acclimated to a standard laboratory diet and tap water under climate-controlled conditions (25 °C, 55% humidity, 12-h light/dark cycle).

### 2.16 Surgical procedures

SD rats (male, n = 56) were randomly assigned to five groups (Fig. S1): (i) autograft (n = 10), (ii) empty conduit (n = 10), (iii) H-scaffold (n = 10), (iv) MGs@H-scaffold (n = 13), and (v) MGs@G-scaffold (n = 13). To assess the *in vivo* survival of transplanted SCs, three additional rats in each of the MGs@H-scaffold and MGs@G-scaffold groups received GFP- SCs@DNM-MGs for fluorescence tracing. All surgical procedures were performed by the same surgeon under sterile conditions using sodium pentobarbital anesthesia (50 mg/kg). An incision was made on the lateral side of the right thigh, and the sciatic nerve was carefully exposed under a surgical microscope. In the autografts group, a 15-mm-long segment of the sciatic nerve was removed, reversed by 180°, and re-implanted. In the empty conduit group, a 15-mm-long sciatic nerve defect was bridged with a size-matched PLLA-co-PTMC conduit. In the H-scaffold, MGs@H-scaffold, and MGs@G-scaffold groups, the nerve defect was bridged using the corresponding size-matched nerve conduits. After implantation, the surgical site was marked with sutures and closed in layers, followed by routine postoperative care including analgesic administration with lidocaine and antibiotic treatment. Additionally, rats in the MGs@H-scaffold and MGs@G-scaffold groups received cyclosporine A (10 mg/kg) according to a standardized administration regimen throughout the experimental period.

### 2.17 Functional recovery evaluation

The nerve functional recovery was assessed by calculating the sciatic functional index (SFI) at 4, 6, 8, 10, and 12 weeks post-surgery. Rats were placed in a confined walkway with a dark shelter at the end, after their hind paws were dipped in red ink. Paw length (PL), toe spread (TS), and intermediary toe spread (IT) were measured from footprints. Data were obtained for both the normal (N) and experimental (E) hind limbs, and the SFI values were determined using the following formula,

$$SFI=-\frac{38.3\left(EPL-NPL\right)}{NPL}+\frac{109.5\left(ETS-NTS\right)}{NTS}+\frac{13.3\left(EIT-NIT\right)}{NIT}-8.8$$

### 2.18 Electrophysiological assessment

Electrophysiological assessments were conducted 12 weeks post-surgery. Five rats from each group were randomly chosen, anesthetized, and their injured sciatic nerves re-exposed. Electrical stimulation was delivered to both the proximal and distal ends of the graft site, and compound muscle action potentials (CMAPs) were recorded from the ipsilateral gastrocnemius muscle using a BL-420F electromyogram recorder (TaiMeng, Chengdu, China). The stimulation parameters were set as follows: pulse mode, stimulus intensity of 10 V, frequency of 1 Hz, and duration of 1 ms. CMAPs amplitudes were determined from the peak voltages of evoked responses. Nerve conduction velocity (NCV) was calculated based on latency differences and the distance between the two stimulating sites (15 mm).

### 2.19 Morphological analysis

Morphological assessment of regenerated nerves was carried out using immunofluorescence staining, toluidine blue staining, and transmission electron microscopy (TEM). Four weeks after surgery, 15-mm nerve segments were harvested from the entire graft region and fixed first in 2.5% glutaraldehyde for 2 h, then in 1% osmium tetroxide for 1.5 h. The samples were subsequently post-fixed in 4% paraformaldehyde for 24 h at 4 °C, dehydrated with 20% (v/v) sucrose overnight, and then submerged in 30% sucrose for an additional 48 h at 4 °C. These dissected tissues were embedded in OCT (Biosharp, China) and cut longitudinally into 10-μm thick cryosections using a freezing microtome (CM1950, Leica, Germany). The sections were permeabilized with 0.5% Triton X-100 in PBS, blocked with 5% BSA in PBS for 1 h, and then incubated overnight at 4 °C with primary antibodies against NF200 (1:150), MBP (1:250), and S100 (1:1000). After rinsing three times with PBS, the sections were incubated with secondary antibodies conjugated to Alexa Fluor 488 anti-Rabbit (1:1000) for NF200 and Alexa Fluor 594 anti-Mouse (1:250) for S100, followed by nuclear counterstaining with DAPI (1:2500) for 20 min. Fluorescence images were captured using a laser scanning confocal microscope (Zeiss, Germany) and analyzed with ImageJ software.

Twelve weeks after surgery, nerve segments (2–3 mm long) were taken from the distal part of the grafts (about 15 mm from the proximal nerve end) and fixed in 2.5% glutaraldehyde for 2 h, followed by 1% osmium tetroxide for 1.5 h. The tissues were dehydrated using a graded ethanol series, embedded in Epon 812 resin (Ted Pella, Redding, CA, USA), and then cut into 1-μm semi-thin slices and 50-nm ultra-thin sections. The semi-thin sections were stained with toluidine blue, and the ultra-thin sections were examined using TEM (JEM-F20, Tokyo, Japan). To quantify the number of myelinated axons, micrographs were captured using a charge-coupled device (CCD) camera (DP70; Olympus, Tokyo, Japan) and analyzed with Image-Pro Plus software (Media Cybernetics, Rockville, MD, USA). Five random fields were analyzed per sample, and five samples from each group were included for statistical evaluation. Myelinated axon diameters and myelin sheath thicknesses were measured with ImageJ software. For each ultrathin section, three axons per field and ten randomly selected fields were analyzed.

The triceps surae muscles were collected from each group 12 weeks after surgery, fixed in 4% paraformaldehyde, and sectioned at 10 μm for Masson's trichrome staining. Micrographs were taken from five random fields per sample with an optical microscope (CX23, Olympus, Japan). The cross-sectional areas of muscle fibers and collagen depositions were measured using ImageJ software. The collagen fiber percentage was determined by dividing the collagen area by the total fibrous area.

### 2.20 Statistical analysis

All statistical analyses were conducted using GraphPad Prism 10 (GraphPad Software, Inc., USA). Data are shown as mean ± SEM (Standard Error of the Mean). Student's t-test was used to compare two groups, while one-way analysis of variance (ANOVA) with Tukey's post hoc test was used to compare three or more groups. Differences were regarded as statistically significant at p < 0.05.

## 3 Results

### 3.1 Fabrication of gradient scaffolds with predefined SCs@DNM-MGs distribution

**Fig. 1:**
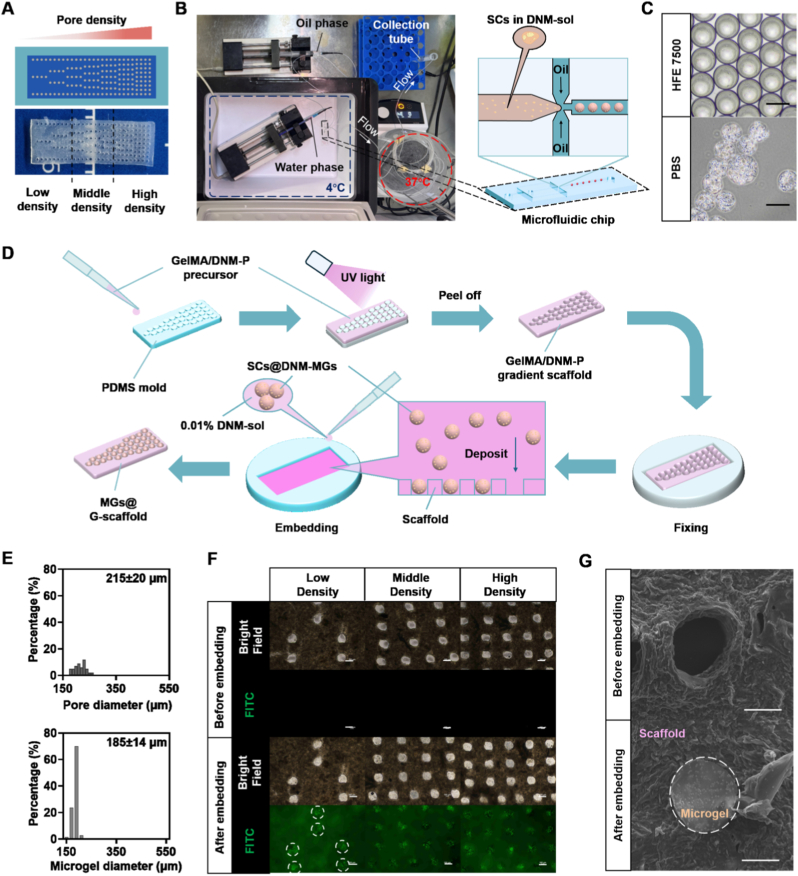
Fabrication and characterization of the MGs@G-scaffold. (A) Schematic illustration (top) and a representative photograph (bottom) of the G-scaffolds, which can be manually divided into three regions along the pore-density gradient direction for subsequent analyses, as indicated by the dashed lines. (B) Top-view image of the MSTC-MS (left), with different temperature zones indicated by colored dashed lines and flow directions marked by white arrows, and the schematic illustration of the microfluidic chip shown on the right. (C) Representative brightfield micrographs showing the SCs@DNM-MGs dispersed in HFE7500 before demulsification and in PBS after demulsification, respectively. Scale bars = 200 μm. (D) Schematic illustration of the fabrication process of the G-scaffold and the subsequent sedimentation of the SCs@DNM-MGs to form MGs@G-scaffold. (E) Measurement of pore diameters in both scaffolds (top) and of the diameters of the SCs@DNM-MGs dispersed in PBS (bottom), with n = 50. (F) Representative brightfield and fluorescence micrographs of the G-scaffold before and after embedding GFP-SCs@DNM-MGs in three designated regions (low, medium, and high densities). The white dashed circles represent the GFP-SCs@DNM-MGs embedded in a G-scaffold. Scale bars = 200 μm. (G) Representative SEM images showing scaffold pores before and after embedding SCs@DNM-MGs. The area outside the circular dashed line represents the scaffold framework, while inside the dashed line indicates SCs@DNM-MGs. Scale bars = 100 μm.

First, photocrosslinkable GelMA was mixed homogeneously with DNM-P to reduce excessive swelling and enhance the stability and bioactivity of the scaffolding material. The resulting GelMA/DNM-P composite scaffold, featuring a long-axis graded pore distribution (i.e., the G-scaffold), was produced through molding using a PDMS replica fabricated from a photolithographically patterned master mold (Fig. 1A). Each pore size in both scaffolds was set to 200 μm. For comparison, a GelMA/DNM-P scaffold with homogeneous pore distribution (i.e., the H-scaffold), but the same pore size and total number of pores as the G-scaffold, was prepared following the same protocol (Fig. S2). On the other hand, primary rat SCs were encapsulated in the DNM microgels using a customized multi-stage temperature-controlling microfluidic system (MSTC-MS) with slight modifications (Fig. 1B). In the MSTC-MS, both oil- and aqueous-phase injectors were connected to a flow-focusing microfluidic chip to emulsify the SCs-loaded DNM solution. To maintain the fluidity of the DNM-sol before gelation, both the aqueous-phase injector and the microfluidic chip were kept at 4 °C. After droplet formation, the emulsified DNM-sol droplets flowed through a silicone tube immersed in a 37 °C water bath to trigger thermal gelation. The resulting SCs@DNM-MGs were highly spherical when collected in the oil phase and retained their shape after demulsification in PBS (Fig. 1C). Quantitative analysis showed that each microgel encapsulated ∼41 ± 5 SCs (Fig. S3), yielding an average loading efficiency of ∼82.4%.

Next, to obtain a gradient distribution of SCs within a single scaffold, SCs@DNM-MGs were resuspended in culture medium with 0.01% DNM solution and applied to the surface of the G-scaffold (Fig. 1D). Due to their relatively high density, the microgels settled by gravity and were individually guided into the pores. The diameters of SCs@DNM-MGs measured 185 ± 14 μm, slightly smaller than the pore sizes of both the G- and H-scaffolds (215 ± 20 μm) (Fig. 1E). Such microsphere size was chosen to balance SC viability, microgel uniformity, and gradient construction efficiency, because excessively large microgels may impair mass transport, whereas overly small microgels would significantly reduce the total number of SCs loaded into the scaffolds. Consequently, the matching sizes and geometric compatibility between the SCs@DNM-MGs and the predefined pores supported the feasibility of achieving single-microgel occupancy within each pore.

As the pore number increased every three rows along the longitudinal axis of the G-scaffold, the three predefined regions exhibited progressively higher local pore densities (low, medium, and high), with a theoretical pore quantity ratio of 11:20:25. Accordingly, each scaffold contained 30, 60, and 75 pores in the low-, medium-, and high-density regions, respectively. This predefined pore-density gradient was designed to establish a spatially organized distribution of SCs@DNM-MGs, thereby generating a biomimetic neurotrophic factor gradient within the scaffold. To verify the efficiency of microgel embedding, primary GFP-SCs were encapsulated within the DNM-MGs before deposition onto the scaffolds. Brightfield and fluorescence microscopy revealed strong GFP fluorescence in nearly all pores across the three regions after microgel sedimentation (Fig. 1F). Notably, the GFP-SC-containing microgels remained stably localized within the pores after subsequent rolling and assembly processes used to fabricate the nerve guidance conduits (Fig. S4). SEM also confirmed the presence of SCs@DNM-MGs inside individual pores (Fig. 1G). These findings collectively show that DNM-MGs are effectively integrated and stably immobilized within the scaffolds, with their distribution matching the designed gradient structure.

### 3.2 DNM-MGs facilitate proliferation and repair-phenotype maintenance of encapsulated SCs

**Fig. 2:**
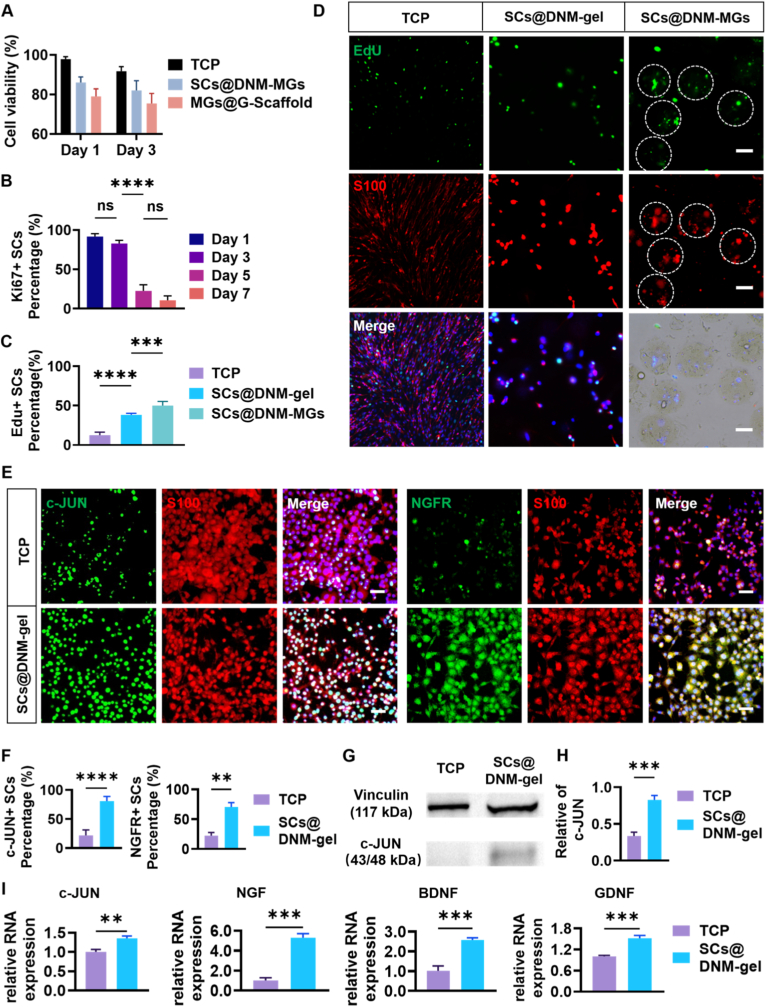
Cytocompatibility, SCs proliferation, and maintenance of the repair phenotype of SCs. (A) Quantitative analysis of cell viability of the SCs cultured on TCP, encapsulated in DNM microgels (SCs@DNM-MGs), or embedded in the MGs@G-scaffold after 1 and 3 days of culture. (B) Proliferation of SCs encapsulated in DNM-MGs assessed by Ki67 immunofluorescence staining on days 1, 3, 5, and 7 of culture. (C) Quantitative analysis and (D) representative fluorescence micrographs of SCs proliferation on TCP, in bulk DNM hydrogel (SCs@DNM-gel), and in SCs@DNM-MGs, assessed by EdU staining after 5 days of culture (n = 5). The white dashed circles indicate the SCs@DNM-MGs. Scale bars = 100 μm. (E) Representative fluorescence micrographs and (F) quantitative analysis of the expression of repair-phenotype markers c-JUN and NGFR in SCs on TCP and in DNM hydrogel (SCs@DNM-gel) after 5 days of culture (n = 5). Scale bars = 50 μm. (G) Representative WB banding patterns and (H) quantitative analysis of c-JUN expression, with Vinculin serving as the loading control. (I) Quantitative real-time PCR (qPCR) analysis of the repair phenotype-related gene c-JUN and paracrine-related neurotrophic factor genes (NGF, GDNF, and BDNF). ∗∗*p* < 0.01, ∗∗∗*p* < 0.001, ∗∗∗∗*p* < 0.0001, and ns represents not significant.

To assess the cytocompatibility of DNM microgels and the composite scaffold, the viability of SCs encapsulated in DNM microgels (SCs@DNM-MGs) and those embedded within the scaffold (MGs@G-scaffold) was evaluated and compared with cells cultured on a tissue culture plate (TCP). Live/dead staining showed that SCs viability in all three groups remained above 75% after 1 and 3 days of culture, demonstrating good cytocompatibility of both the DNM microgels and the scaffold (Fig. 2A, Fig. S5). Cell proliferation is a key feature of the repair phenotype of SCs. Therefore, SCs@DNM-MGs cultured for 1, 3, 5, and 7 days were stained using the cell-proliferation marker Ki67. A significant decrease in the percentage of Ki67+ SCs was observed after five days, with an even lower percentage on Day 7, indicating that encapsulated SCs exited the rapid proliferation phase after extended culture (Fig. 2B, Fig. S6). Based on this observation, the *in vitro* pre-culture period for SCs@DNM-MGs was limited to no more than 5 days prior to *in vivo* transplantation. To further compare proliferative activity, SCs encapsulated in bulk DNM gel (SCs@DNM-gel) and DNM microgels were examined through immunostaining using the proliferation marker EdU, with TCP-cultured SCs serving as controls (Fig. 2C and D). SCs@DNM-MGs showed a significantly higher proportion of EdU + SCs compared to SCs@DNM-gel. Meanwhile, SC@DNM-gel showed increased proliferation compared to the TCP group. This suggests that both the tissue-specific bioactivity of DNM and the improved mass-transport efficiency of the microgels promote SC proliferation.

Furthermore, the expression of phenotype-associated markers c-JUN and NGFR (p75 neurotrophin receptor) was examined using immunofluorescence staining (Fig. 2E). The proportions of the c-JUN+ and NGFR + SCs identified in the SCs@DNM-gel were significantly higher than those in the TCP group (Fig. 2F). Consistently, Western blot (WB) analysis confirmed an increase in the key c-JUN protein expression in the SCs cultured in DNM-gel compared with TCP (Fig. 2G and H), indicating that DNM supports the maintenance of the repair phenotype at the protein level. Considering that the paracrine activity of SCs is concurrently augmented during the adoption of the repair phenotype, the secretion of neurotrophic factors (NGF, BDNF, and GDNF) by SCs@DNM-MGs was quantified through the analysis of culture supernatants using ELISA after five days (Fig. S7). Concentrations of all three neurotrophic factors in the supernatants of SCs@DNM-gel were significantly higher than those in TCP, indicating enhanced paracrine function of SCs when encapsulated within DNM-MGs. Additionally, qRT-PCR analysis showed increased transcription of repair-phenotype and paracrine-related genes in SCs cultured three-dimensionally within the DNM-gel (Fig. 2I), including c-JUN, NGF, BDNF, and GDNF, further confirming the supportive role of DNM in maintaining the repair phenotype of SCs at the transcriptional level. Overall, these findings suggest that DNM microgel encapsulation enhances SC proliferation and stabilizes their repair phenotype. These preconditioned SCs can serve as reliable cellular units for transplantation and provide an essential cellular foundation for developing a sustained, long-term neurotrophic gradient within the engineered scaffold.

### 3.3 MGs@G-scaffold constructs a sustained gradient of neurotrophic factors that guide axonal regeneration

**Fig. 3:**
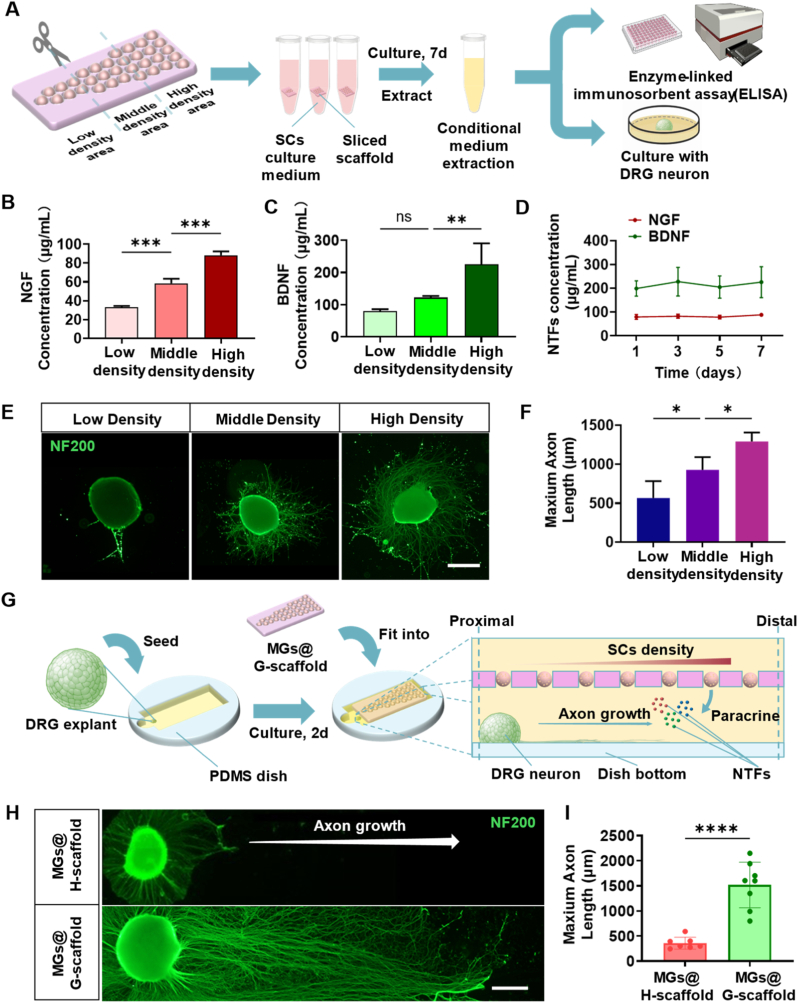
Generation of the NTF gradient through predefined SCs distribution by immobilized SCs@DNM-MGs and its effects on axonal growth. (A) Schematic illustration of the experimental strategy for analyzing the NTF gradient within the MGs@G-scaffold. The scaffold was divided into three regions along the longitudinal axis for further analysis. (B) NGF and (C) BDNF concentrations in culture supernatants collected from the three regions of the MGs@G-scaffold after 7 days of culture. (D) Time-dependent release profiles of NGF and BDNF from the high SCs density region of the MGs@G-scaffold at 1, 3, 5, and 7 days. (E) Representative fluorescence micrographs and (F) quantitative analysis of DRG explants cultured for 7 days in conditioned media derived from the three regions of the MGs@G-scaffold, assessed by NF200 immunostaining (n = 4). Scale bars = 200 μm. (G) Schematic illustration of an *in vitro* DRG model developed to study how NTF gradients influence axonal regeneration. (H) Representative fluorescence images and (I) quantitative analysis of DRG neurons after 14 days of co-culture with MGs@H-scaffold and MGs@G-scaffold, respectively, assessed by NF200 immunostaining (n = 8). ∗*p* < 0.05, ∗∗*p* < 0.01, ∗∗∗*p* < 0.001, ∗∗∗∗*p* < 0.0001, and ns represents not significant.

To verify the distribution of neurotrophic factors (NTFs) produced by the secretion of immobilized SCs@DNM-MGs, the MGs@G-scaffold was manually cut into three predefined regions along its long axis, including low, medium, and high densities, as shown in Fig. 1A, and cultured in SCs medium for 7 days (Fig. 3A). Cultural supernatants from each region were collected separately for subsequent ELISA quantification and used as conditional media for DRG culture. First, ELISA analysis shows that both NGF and BDNF concentrations gradually decrease from the high-density area to the low-density area along the long axis of the MGs@G-scaffold (Fig. 3B and C), confirming the development of a spatial gradient in NTF distribution. Additionally, daily release profiles showed sustained secretion of both NGF and BDNF over a week, with no significant decreases (Fig. 3D), indicating stable, continuous paracrine activity of the encapsulated SCs. Meanwhile, the conditioned media collected from the three regions were applied to DRG cultures for 7 days. Immunofluorescence staining for neurofilament (NF200) showed that conditioned medium derived from the high-density region significantly improved axonal extension compared with medium from either the middle- or low-density region (Fig. 3E). Statistical analysis confirmed that the axon lengths of the cultured DRG neurons increase proportionally with higher densities of the immobilized SCs@DNM-MGs (Fig. 3F). This dose-dependent response suggests that the spatial gradient distribution of SCs@DNM-MGs within the MGs@G-scaffold directly translates into graded neurotrophic bioactivity.

To further explore how the NTF gradient generated by MGs@G-scaffold can steer axonal growth directionally, a DRG-based *in vitro* model was established. A DRG explant was placed next to the low-density side of the MGs@G-scaffold to mimic proximal neurons extending axons toward a distant target (Fig. 3G). As a control, the gradient scaffold was replaced with the MGs@H-scaffold to diminish spatial variation in NTF concentrations. After 14 days of culture, the DRG cultured with the MGs@G-scaffold showed highly oriented and significantly longer outgrowing axons toward the distal region, compared to those cultured with the MGs@H-scaffold (Fig. 3H and I). These results demonstrate that the gradient-immobilized SCs@DNM-MGs created a proximal-to-distal, self-maintained NTF gradient around the MGs@G-scaffold. This cell-mediated biochemical gradient provides sustained guidance cues for axonal extension, highlighting its strong potential to promote long-distance nerve regeneration and functional recovery *in vivo*.

### 3.4 MGs@G-scaffold transplantation promotes axonal regeneration

**Fig. 4:**
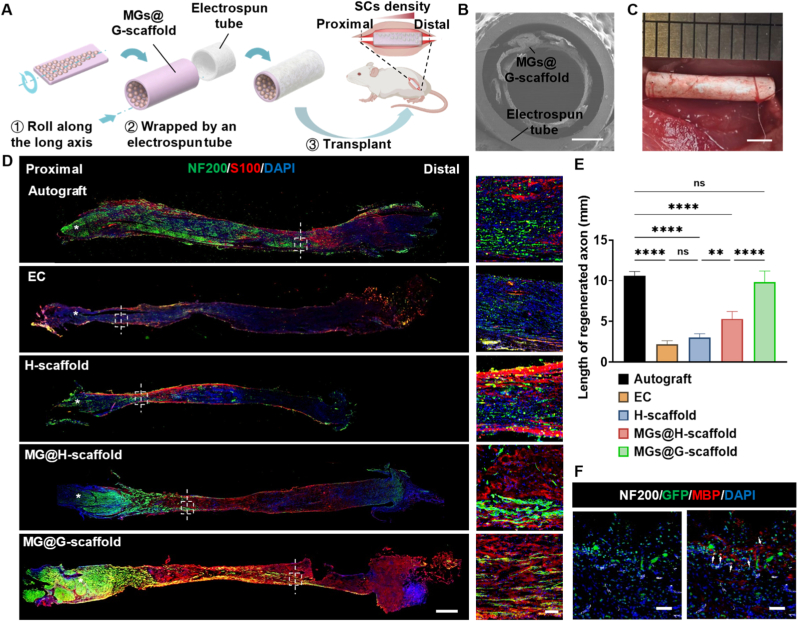
Nerve graft fabrication, transplantation, and axonal regeneration after treatment of sciatic nerve defect in rats. (A) Schematic illustration of the fabrication process and transplantation of a nerve graft containing MGs@G-scaffold. (B) Cross-sectional SEM micrograph of an electrospun conduit loaded with the MGs@G-scaffold. Scale bar = 500 μm. (C) Surgical transplantation of the nerve conduit into a rat sciatic nerve defect model. A 15-mm nerve conduit was used to bridge the transected sciatic nerve. (D) Representative immunofluorescence micrographs of the longitudinal sections from each experimental group, four weeks post-surgery. Axons and SCs were identified by NF200 (green) and S100 (red), respectively, with DAPI used for nuclear counterstaining. Scale bar = 1 mm (macroscopic view) and 100 μm (magnified view). (E) Quantitative analysis of the length of regenerated axons in each grafted group, n = 5. ∗∗*p* < 0.01, ∗∗∗∗*p* < 0.0001, and ns represents not significant. (F) Representative immunofluorescence micrographs of longitudinal sections from the MGs@G-scaffold group four weeks post-surgery. Regenerated axons, implanted SCs, and myelinated SCs were labeled with NF200 (white), GFP (green), and MBP (red), respectively, with DAPI used for nuclei counterstaining. White arrows indicate colocalization of GFP and MBP signals, suggesting differentiation of implanted SCs into myelinating SCs. Scale bar = 50 μm. (For interpretation of the references to color in this figure legend, the reader is referred to the Web version of this article.)

The implantable nerve grafts were fabricated by rolling the MGs@G-scaffold and MGs@H-scaffold lengthwise, then inserting them into electrospun PLLA-co-PTMC tubes (Fig. 4A), which exhibited good resistance to compression, maximized resistance to surgical suture tension and compression from surrounding tissues. In the grafts loaded with MGs@G-scaffolds, the low-density region was sutured to the proximal stump of a transected nerve, thereby establishing an increasing NTF gradient along the direction of axonal extension. SEM characterization showed that the rolled MGs@G-scaffold maintained a hollow lumen structure inside the electrospun conduit, which provided a permissive space for regenerating axons to traverse the injured site (Fig. 4B). The sciatic nerve autografts (the Autograft group), empty PLLA-co-PTMC conduits (the EC group), and PLLA-co-PTMC conduits containing H-scaffolds without SCs@DNM-MGs (the H-scaffold group) were used as controls. These were transplanted into a 15-mm sciatic nerve defect model in rats, along with those containing either MGs@H-scaffolds (the MGs@H-scaffold group) or MGs@G-scaffolds (the MGs@G-scaffold group, as shown in Fig. 4C).

Four weeks after surgery, the regenerated nerves within the grafts were examined by immunofluorescence staining using NF200 and S100 (Fig. 4D). It was observed that the EC group showed minimal nerve regeneration, with only sparse NF200+ axons and S100+ SCs detected within the lesion, likely due to a lack of structural and biochemical support. Implantation of the H-scaffold conduits provided physical guidance for cellular adhesion and infiltration, but the length of regenerated axons did not differ significantly from that in the EC group (Fig. 4E). In contrast, both the MGs@H-scaffold and MGs@G-scaffold demonstrated significantly greater axonal extension compared to the EC and H-scaffold groups. These findings suggest that transplanting exogenous SCs and their paracrinely produced NTFs offers crucial biochemical support for early nerve regeneration. Notably, when the transplanted SCs were arranged in a predefined gradient within the nerve graft, the MGs@G-scaffold showed significantly longer regenerated axons and a more aligned distribution of axons and SCs compared to the MGs@H-scaffold group. Importantly, the MGs@G-scaffold demonstrated the ability to promote axonal extension comparable to that of the Autograft group at the early stage. To examine the fate of implanted SCs at the same time point, regenerated nerves and myelinated SCs were co-immunostained using NF200 and MBP. This was tested on specific MGs@G-scaffolds with GFP-SCs@DNM-MGs (Fig. 4F). A significant number of exogenous GFP-SCs were found within the graft, some of which colocalized with MBP around axons, indicating that the implanted SCs survived and were involved in myelination guided by the regenerating nerves.

### 3.5 MGs@G-scaffold facilitates nerve remyelination

**Fig. 5:**
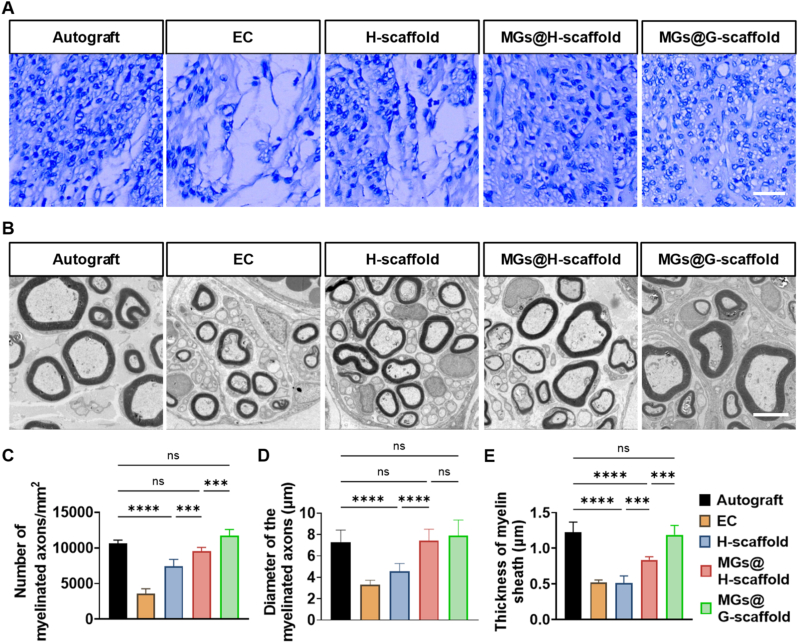
Remyelination of regenerated axons in all grafted nerves twelve weeks post-surgery. (A) Representative transverse sections of the harvested nerve grafts stained with toluidine blue. Scale bar = 20 μm. (B) Representative TEM micrographs of myelinated axons in the distal portion of the grafts. Scale bar = 5 μm. Quantitative analysis of (C) the number of myelinated axons, (D) the diameter of the myelinated axons, and (E) the thickness of the myelin sheath, n = 5. ∗∗∗*p* < 0.001, ∗∗∗∗*p* < 0.0001, and ns represents not significant. (For interpretation of the references to color in this figure legend, the reader is referred to the Web version of this article.)

To further evaluate the degree of maturation of the regenerated nerve fibers, myelination within the nerve grafts was examined twelve weeks after surgery. First, histological analysis showed a significantly higher density of myelinated axons in grafts with transplanted SCs, including both MGs@H-scaffold and MGs@G-scaffold, compared with the EC and H-scaffold groups, as shown by toluidine blue staining (Fig. 5A). This suggests that the presence of exogenous SCs greatly improved remyelination of the regenerated axons. It was observed that the gradient distribution of SCs further promoted the myelination of regenerated axons, as demonstrated by a significantly higher number of myelinated nerve fibers in the MGs@G-scaffold group compared to the MGs@H-scaffold group (Fig. 5C). Further TEM analysis was performed to measure the diameter of myelinated axons and the thickness of the myelin sheath. Well-formed myelin sheaths with electron-dense lamellar structures were observed in all graft groups after twelve weeks (Fig. 5B). Among them, the Autograft group showed the largest axonal diameter and the thickest myelin sheaths, indicating the superior regenerative capacity of the autologous nerve grafts. While the SCs-containing grafts, i.e., MGs@H-scaffold and MGs@G-scaffold, exhibited similar myelin structure integrity and axonal diameter as the Autograft group (Fig. 5D and E). The MGs@G-scaffold group showed a significantly thicker myelin sheath compared to the MGs@H-scaffold group. Overall, these results indicate that the predefined SCs gradient within the MGs@G-scaffold not only accelerated axonal growth but also promoted remyelination and maturation of the regenerated nerve fibers.

### 3.6 MGs@G-scaffold improves nerve functional recovery

**Fig. 6:**
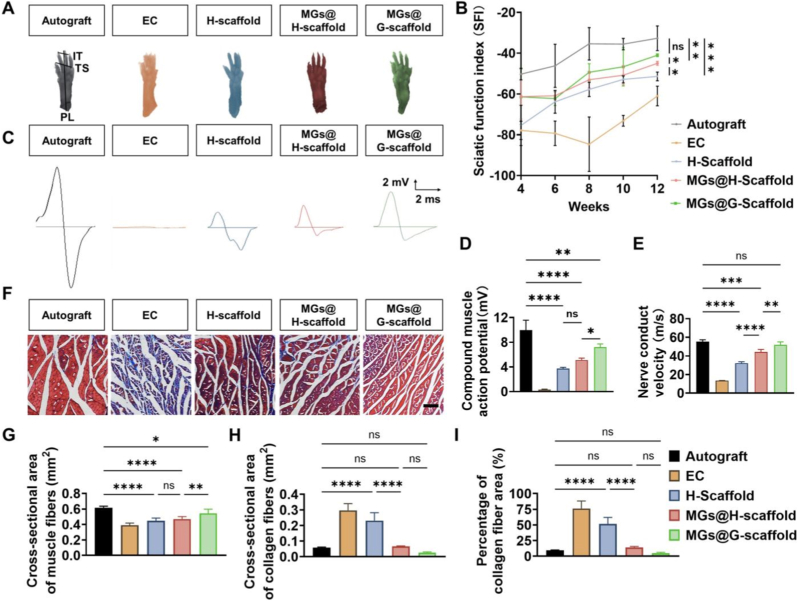
Assessments of sciatic nerve functional recovery following transplantation of engineered grafts. (A) Representative footprints of the injured hind limbs were used for SFI analysis twelve weeks after surgery. Paw length (PL), toe spread (TS), and intermediary toe spread (IT) from both the injured and contralateral normal limbs were measured. (B) Quantitative analysis of SFI values at 4, 6, 8, 10, and 12 weeks post-surgery (n = 5). (C) Representative CMAP recordings from the regenerated nerves in each experimental group. Quantitative analyses of both (D) CMAP amplitudes and (E) NCV values across all groups. (F) Representative cross-sectional images of the triceps surae muscles harvested twelve weeks post-surgery, displayed with Masson's trichrome staining. Scale bar = 100 μm. Quantitative analyses of (G) muscle fiber area, (H) collagen fiber area, and (I) percentage of collagen fiber area (n = 4). ∗*p* < 0.05, ∗∗*p* < 0.01, ∗∗∗*p* < 0.001, ∗∗∗∗*p* < 0.0001, and ns represents not significant.

As an indicator of motor function recovery, sciatic functional index (SFI) values, ranging from 0 (normal function) to −100 (complete dysfunction), were calculated from rat footprints recorded every two weeks post-surgery (Fig. 6A). Progressive increases in SFI values were observed in all groups over time, indicating the gradual restoration of motor nerve function. As expected, the Autograft group consistently showed the highest SFI values throughout the entire observation period (Fig. 6B). After twelve weeks, the MGs@G-scaffold group showed a significantly higher SFI value than all the EC, H-scaffold, and MGs@H-scaffold groups, demonstrating that the gradient distribution of transplanted SCs greatly enhanced motor function recovery.

Electrophysiological analysis was also conducted twelve weeks after transplantation to assess nerve conduction across the nerve grafts (Fig. 6C). The Autograft group showed the highest compound muscle action potential (CMAP) amplitude and nerve conduction velocity (NCV). Notably, the NCV value of the MGs@G-scaffold group was similar to that of the Autograft group, while its CMAP amplitude was the highest among all engineered grafts, indicating its superior ability to restore nerve conduction function (Fig. 6D and E). Although the MGs@H-scaffold group showed a smaller CMAP amplitude than the MGs@G-scaffold group, it demonstrated a much higher NCV than the EC and H-scaffold groups, emphasizing the importance of exogenous SC transplantation.

To further assess the recovery of target muscles, Masson's trichrome staining was performed on the triceps surae muscles harvested from the operated limbs (Fig. 6F, Fig. S8). The Autograft group maintained the largest muscle fiber area but showed minimal collagen deposition (Fig. 6G–I), indicating the most effective preservation of muscle integrity. Meanwhile, the MGs@G-scaffold group exhibited a significantly larger cross-sectional area of muscle fibers and considerably less collagen accumulation compared with the other implanted engineered grafts. Furthermore, both MGs@G-scaffold and MGs@H-scaffold showed significantly lower collagen deposition than the H-scaffold group, highlighting the essential role of the exogenous SCs in reducing muscle atrophy. These findings demonstrate that the transplanted SCs with a spatially gradient distribution effectively improved nerve functional recovery and supported the preservation of downstream muscle after severe peripheral nerve transection.

## 4 Discussion

It is well-known that SCs play a vital role in peripheral nerve regeneration. They can dedifferentiate and migrate to form Büngner bands, which provide physiological guidance for axonal extension and eventually contribute to remyelination [4]. Furthermore, dedifferentiated SCs secrete multiple NTFs, including NGF, BDNF, and GDNF, supporting axon elongation in a sustainable manner [32]. However, severely transected PNI often causes significant dysfunction or even apoptosis of endogenous SCs [33]. Therefore, transplanting exogenous SCs is highly important for PNI repair, especially in cases of long-distance transected injuries. In our *in vivo* assessments, it was noticed that all transplanted nerve grafts loaded with exogenous SCs, including both MGs@H-scaffolds and MGs@G-scaffolds, showed superior performance compared to cell-free scaffolds, in terms of axonal extension (Fig. 4D and E), remyelination (Fig. 5), and functional recovery (Fig. 6), which underscores the importance of SC transplantation.

Recently, many research studies have reported that both endogenous and exogenous SCs spontaneously undergo senescence and lose their regenerative capacity during long-term PNI repair [[34], [35], [36]]. Therefore, maintaining the repair phenotype in transplanted SCs is crucial for successful nerve regeneration. In this study, DNM-based microgels were used as modular carriers for SC encapsulation and integration into engineered nerve grafts. The DNM-MGs not only provided physical protection during handling and transplantation but also created a tissue-specific microenvironment that supported SC survival and proliferation (Fig. 2A–D, Fig. S6) and preserved their repair phenotype (Fig. 2E–H). Notably, a highly elevated expression of repair-associated genes (e.g., c-JUN, NGF, BDNF, and GDNF) was observed in the SCs@DNM-MGs (Fig. 2I), along with increased secretion of NTFs (Fig. S7). This effect is likely due to the preservation of native extracellular matrix components within the DNM, which provide biochemical cues that mimic the niches of peripheral nerves *in vivo* [37]. Additionally, the high mass-transport efficiency of the microgels facilitates nutrient and oxygen exchange and waste removal. Furthermore, GFP-SCs transplanted via DNM-MGs were found to survive and contribute to myelination during nerve regeneration at 4 weeks post-surgery, indicating their continued functional involvement in tissue repair (Fig. 4F). Together, these features enabled the efficient delivery of a large number of functionally active SCs while maintaining their pro-regenerative phenotype.

On the other hand, our transplanted SCs not only provide permissive guidance tracks but also secrete various NTFs in a sustained manner, serving as biochemical cues for nerve regeneration. However, growing evidence shows that it is not just the NTFs but their spatial gradients that play a central role in guiding cell migration and axon extension. Soluble gradients of biological NTFs, such as NGF, BDNF, and GDNF, can activate TrkA/PI3K/Akt, Ras/MAPK, and RhoA/ROCK signaling pathways to promote axonal elongation from lower to higher concentrations by polymerizing actin filaments and stabilizing microtubules [[38], [39], [40]]. Traditional methods for creating biochemical gradients in nerve grafts mainly depend on incorporating and controlling the release of exogenous NTFs. Various strategies, such as heparin-mediated binding, chemical crosslinking, and multilayer deposition within polymer matrices, have been used to immobilize growth factors and control their release rates [[41], [42], [43]]. However, these methods often involve complex fabrication or chemical modifications, which can reduce growth factor bioactivity and loading efficiency. Additionally, the type of NTFs incorporated into the grafts was predetermined, so the complex requirements for directed axonal growth have yet to be met, and achieving temporally coordinated release of multiple factors remains challenging.

In contrast, cell-based strategies provide a dynamic and self-regulating method for neurotrophic support. Transplanted cells, including SCs, mesenchymal stem cells, and neural progenitors, can continuously secrete multiple NTFs in response to the local microenvironment, thus offering more physiologically relevant and sustained biochemical cues. Importantly, these strategies address issues related to protein instability and the loss of bioactivity during processing [[44], [45], [46]]. So far, multiple fabrication techniques have been developed to spatially arrange therapeutic cells within tissue-engineered nerve grafts. These include gradient bioprinting, layer-by-layer assembly, and cell self-organization guided by chemokines or capillaries [[47], [48], [49]]. For example, a recent study showed that longitudinal-gradient porous structures can induce distal enrichment of SCs via the capillary effect, thereby mimicking the endogenous regenerative microenvironment after peripheral nerve injury [50]. Despite these advances, precisely organizing cells in space while maintaining a favorable bioactive microenvironment for transplanted SCs remains technically challenging. Our strategy employed the modular assembly of SCs@DNM-MGs, enabling a predetermined spatial distribution of SCs while preserving tissue-specific extracellular matrix components and the high mass-transport properties of the microgels. These features have been shown to enhance SC survival, preserve their repair-associated bioactivity, and promote sustained paracrine secretion of neurotrophic factors, thereby facilitating the establishment of physiologically relevant neurotrophic gradients within the graft.

In this study, by integrating DNM-MGs with a predefined, gradient-based cellular distribution, our engineered MGs@G-scaffold established a proximal-distal NTF gradient by controlling the regional number of NTF-secreting SCs, effectively promoting directional axonal growth *in vitro* (Fig. 3). Previous studies have demonstrated that even a mild NTF gradient can promote directional axonal extension by asymmetrically activating neurotrophin receptor-mediated signaling in growth cones, while also facilitating SC chemotactic migration and Büngner band formation [51]. In addition, the spatial distribution of NTFs may contribute to stage-dependent regulation of SC behaviors during regeneration, including maintenance of repair-associated phenotypes in the proximal region and promotion of remyelination in the distal region following axonal extension. Given that the MGs@H-scaffold and MGs@G-scaffold exhibited comparable total SC counts, the improved regeneration observed in the MGs@G-scaffold is more plausibly ascribed to the spatially organized SC distribution and the consequent biomimetic NTF gradient, rather than to a mere effect of SC transplantation. Moreover, all forms of DNM-containing hydrogels can serve as an alternative platform for growth factor loading because they preserve bioactive components like heparan sulfate proteoglycans. Compared to the homogeneous cell distribution in MGs@H-scaffold, the gradient organization led to significantly longer axonal growth and more aligned axon-SC architecture *in vivo*, as well as improved directional neurite outgrowth in DRG co-culture.

Overall, these findings show that biomaterial-assisted transplantation of spatially organized, repair-phenotype SCs offers a combined approach that merges sustained biochemical signaling with functional cellular support. This approach not only overcomes the limitations of traditional growth factor delivery systems but also emphasizes the importance of spatially defined cell organization in guiding long-distance peripheral nerve regeneration. Despite the promising therapeutic outcomes, two limitations of the present study should be noted. First, although the MGs@G-scaffold successfully established a sustained NTF gradient through spatially distributed SCs, the relationship among SC distribution patterns, NTF gradient profiles, and regenerative efficacy remains to be systematically optimized [21]. Second, although SCs encapsulated in DNM-MGs exhibited robust repair-associated phenotypes *in vitro*, the long-term phenotypic stability of transplanted SCs *in vivo* remains unclear and warrants further investigation [8,52].

## 5 Conclusion

In this study, an engineered nerve graft with gradient-distributed, immobilized SCs@DNM-MGs was developed for cell transplantation and PNI repair. The DNM-MGs provided a regenerative-supportive microenvironment that maintained the high viability, proliferative capacity, and repair phenotype of the encapsulated SCs, thereby ensuring their functional bioactivity before and after scaffold transplantation. By leveraging the paracrine effect of the exogenous SCs, this system establishes a stable, self-sustained gradient of secreted NTFs, including NGF, BDNF, and GDNF. The gradient scaffold not only accelerates axonal extension but also facilitates the remyelination and maturation of regenerated nerve fibers, ultimately resulting in significantly enhanced electrophysiological performance and functional recovery *in vivo*. It has been demonstrated that the transplantation of spatially graded SCs is pivotal in promoting long-distance peripheral nerve regeneration. Overall, this study presents a versatile and efficacious strategy for developing cell-instructive nerve grafts and offers new perspectives on the design of tissue-engineered scaffolds for regenerative medicine.

## CRediT authorship contribution statement

**Shengwen Zhu:** Data curation, Formal analysis, Investigation, Methodology, Visualization, Writing – original draft. **Rui Cui:** Data curation, Formal analysis, Investigation, Methodology, Validation, Visualization. **Shuai Qiu:** Data curation, Investigation, Methodology, Resources, Validation. **Xi Zhang:** Data curation, Investigation. **Jingxin Ma:** Investigation, Validation. **Wan Duan:** Investigation, Validation. **Peiyao Li:** Investigation, Validation. **Daping Quan:** Investigation, Resources. **Zehong Yang:** Funding acquisition, Resources, Validation. **Sien Zhang:** Resources, Validation. **Zilong Rao:** Data curation, Funding acquisition, Investigation, Methodology, Resources, Validation, Writing – review & editing. **Ying Bai:** Conceptualization, Funding acquisition, Project administration, Resources, Supervision, Validation, Writing – review & editing.

## Declaration of competing interest

The authors declare that they have no known competing financial interests or personal relationships that could have appeared to influence the work reported in this paper.

## Data Availability

Data will be made available on request.
