# Supplementary material for: All-in-one nerve guidance: transplanting a gradient scaffold with immobilized Schwann cells for peripheral nerve regeneration
Source: Mater Today Bio. 2026 May 28;38:103294. doi: 10.1016/j.mtbio.2026.103294 (PMC13241973; doi:10.1016/j.mtbio.2026.103294)
Supplement: Multimedia component 1 [file mmc1.docx]

Supporting Information

**All-in-one nerve guidance: transplanting a gradient scaffold with immobilized Schwann cells for peripheral nerve regeneration**

Shengwen Zhu ^a,1^, Rui Cui ^a,1^, Shuai Qiu ^b^, Xi Zhang ^a^, Jingxin Ma ^c^, Wan Duan ^c^, Peiyao Li ^c^, Daping Quan ^a^, Zehong Yang ^d^, Sien Zhang ^c^, Zilong Rao ^a,^ *, Ying Bai ^a,^ **

^a^ *Guangdong Engineering Technology Research Centre for Functional Biomaterials, Key Laboratory for Polymeric Composite & Functional Materials of Ministry of Education, School of Materials Science and Engineering, Sun Yat-sen University, Guangzhou 510006, China*

^b^ *Department of Orthopedics, The Eighth Affiliated Hospital, Sun Yat-sen University, Shenzhen, 518033, China*

^c^ *Hospital of Stomatology, Guanghua School of Stomatology, Sun Yat-sen University, Guangdong Provincial Key Laboratory of Stomatology, Guangzhou, 510055, China*

^d^ *Department of Radiology, Sun Yat-Sen Memorial Hospital, Sun Yat-Sen University, Guangzhou, 510120, China*

* Corresponding author.

** Corresponding author.

*Email addresses:* raozlong@mail.sysu.edu.cn (Z. Rao), baiy28@mail.sysu.edu.cn (Y. Bai).

^1^ These authors contributed equally to this work.


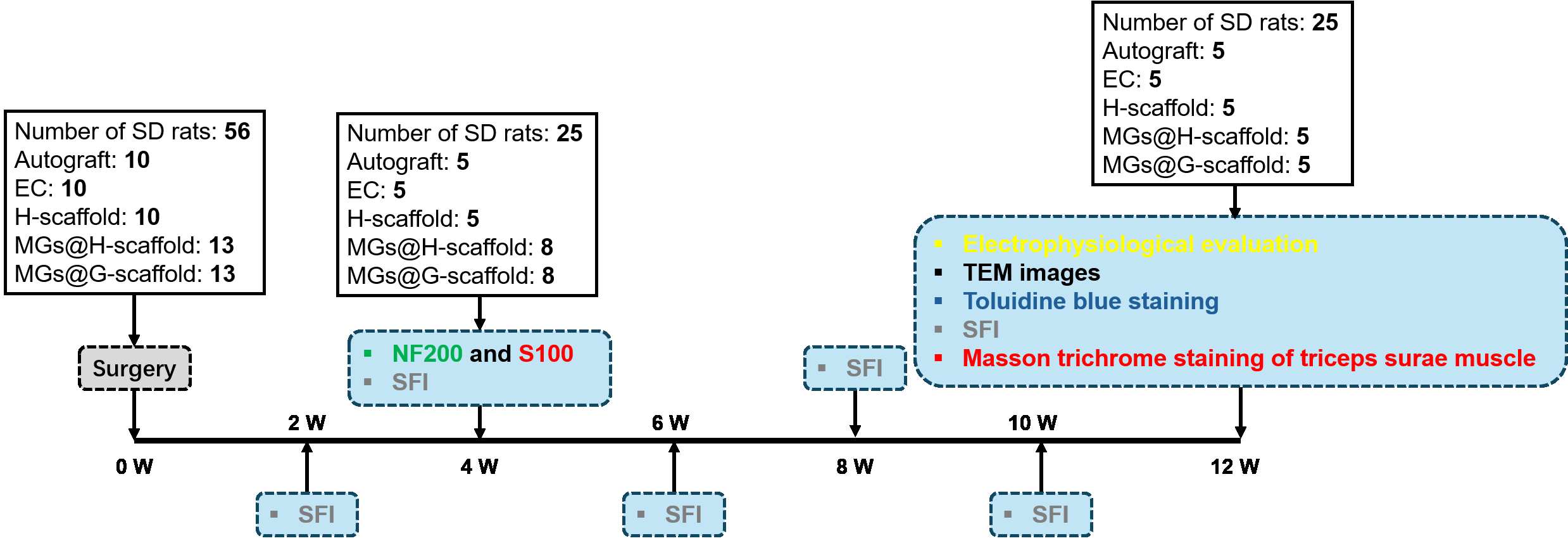


**Fig. S1.** Diagram of the experimental setup, including the number of rats and their specific purposes in each group.


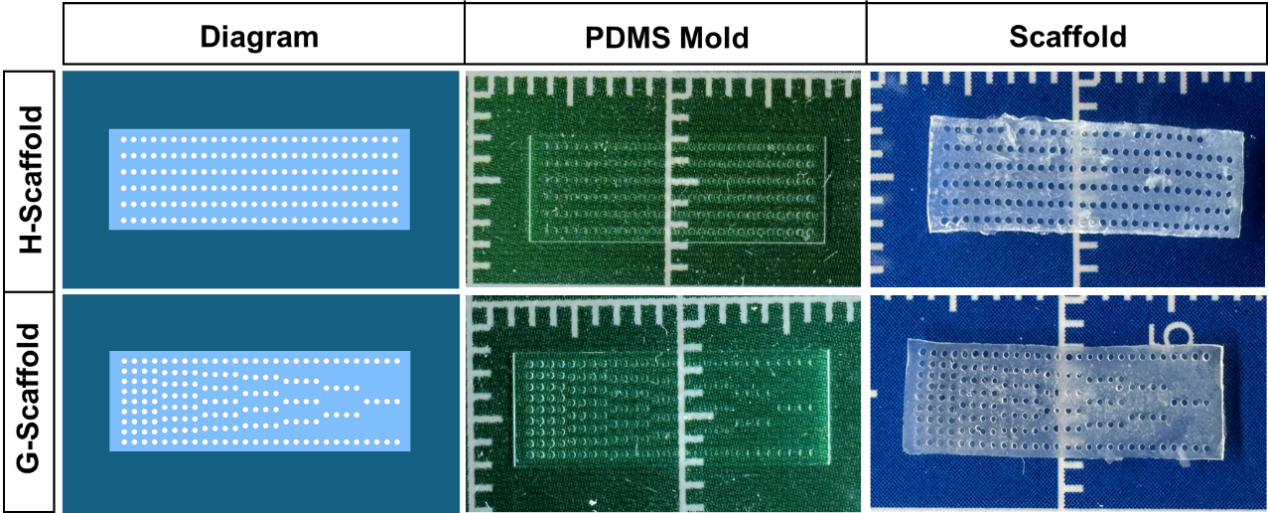


**Fig. S2.** Two master molds (diagrams on the left) were designed with uniformly distributed circles for the H-scaffold and varying numbers of circles for the G-scaffold. The PDMS precursor was poured into both master molds, creating PDMS molds with predefined column-like protrusions (as shown in the representative photographs in the middle). Finally, the H-scaffold and G-scaffold were fabricated with uniform and gradient pore distributions, respectively, by molding GelMA/DNM-P composite hydrogel into the PDMS molds (as shown in the representative photographs on the left).


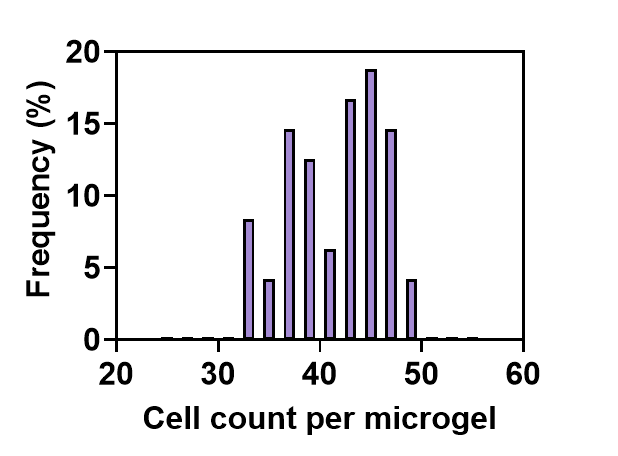


**Fig. S3.** Distribution of SC numbers encapsulated within individual SCs@DNM-MGs, as determined by bright-field microscopy.

**
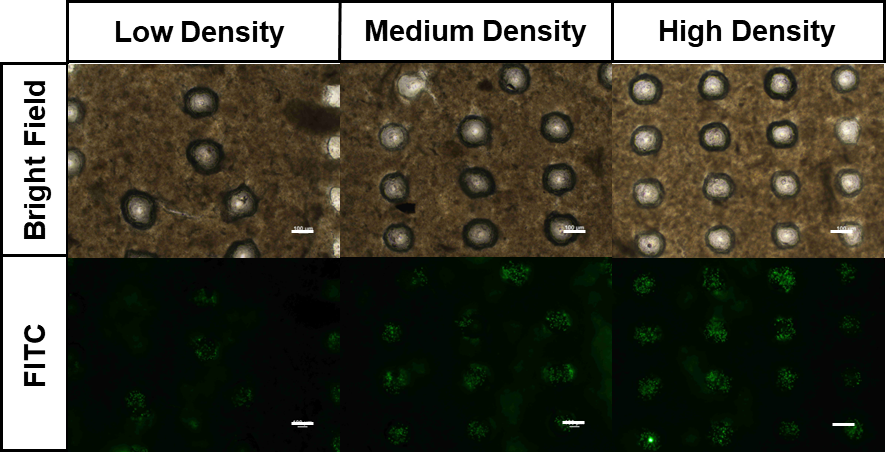
**

**Fig. S4.** Representative brightfield and fluorescence micrographs of the MGs@G-scaffold loaded with GFP-SCs@DNM-MGs after sequential washing, longitudinal rolling, and unrolling. GFP-SCs@DNM-MGs remained stably localized within the pores of the G-scaffold. Scale bars = 200 μm.


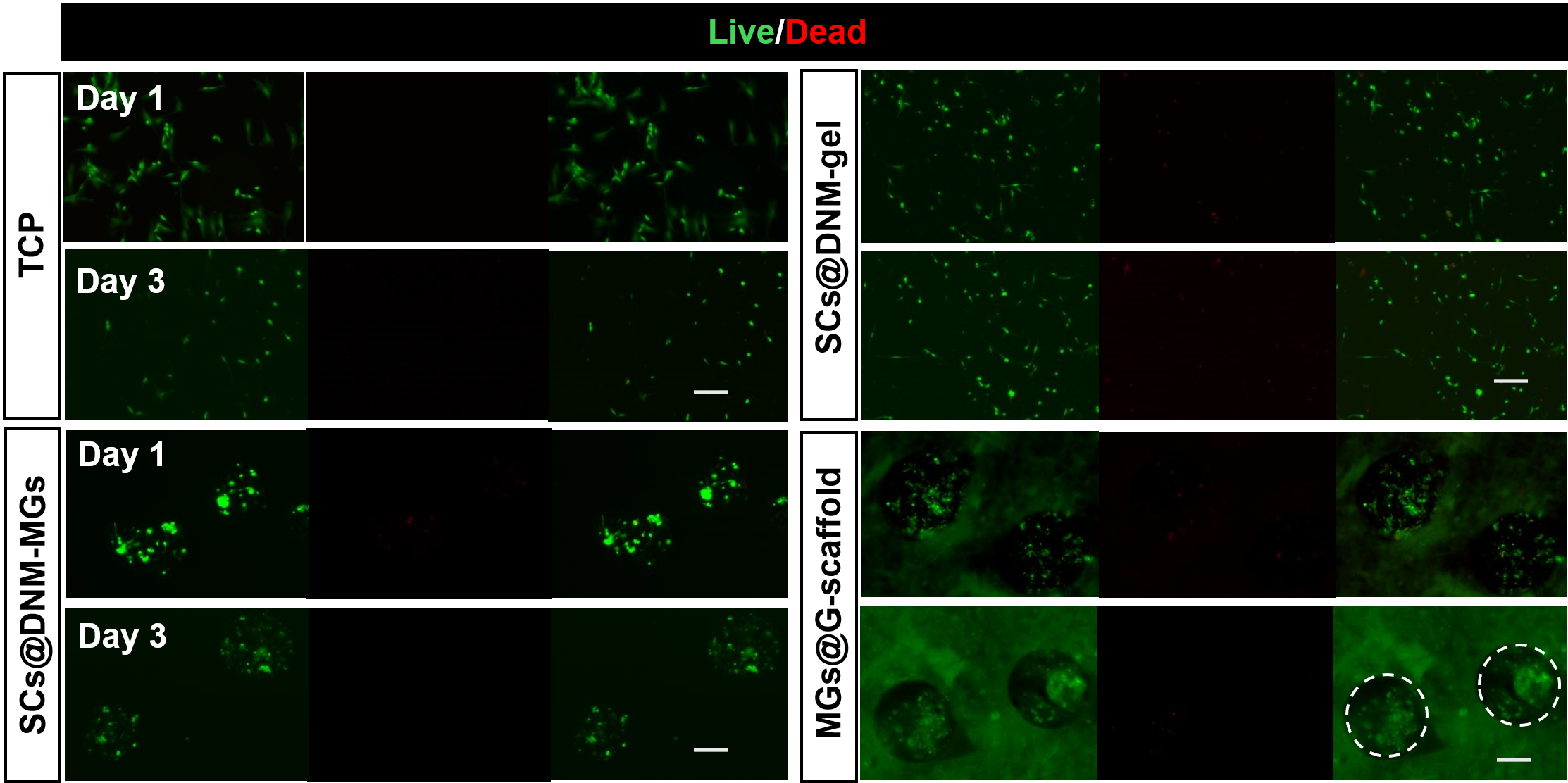


**Fig. S5.** Representative fluorescence micrographs of live/dead stained TCP, SCs@DNM-gel, SCs@DNM-MGs, and MGs@G-scaffold after one and three days of culture, respectively. The white dashed circles represent the embedded SCs@DNM-MGs within the scaffold.


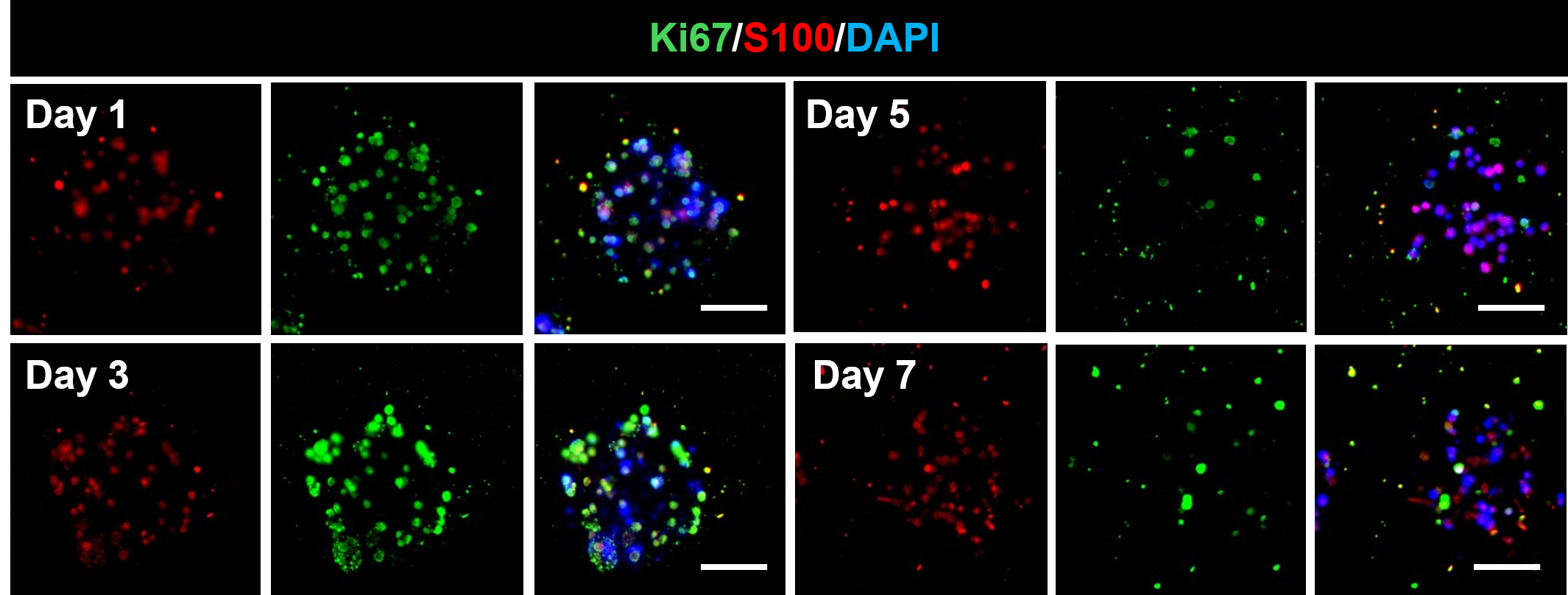


**Fig. S6.** Representative fluorescence micrographs of the SCs@DNM-MGs after one and three days of culture, immunostained with Ki67 and S100, respectively. Scale bars = 100 μm.


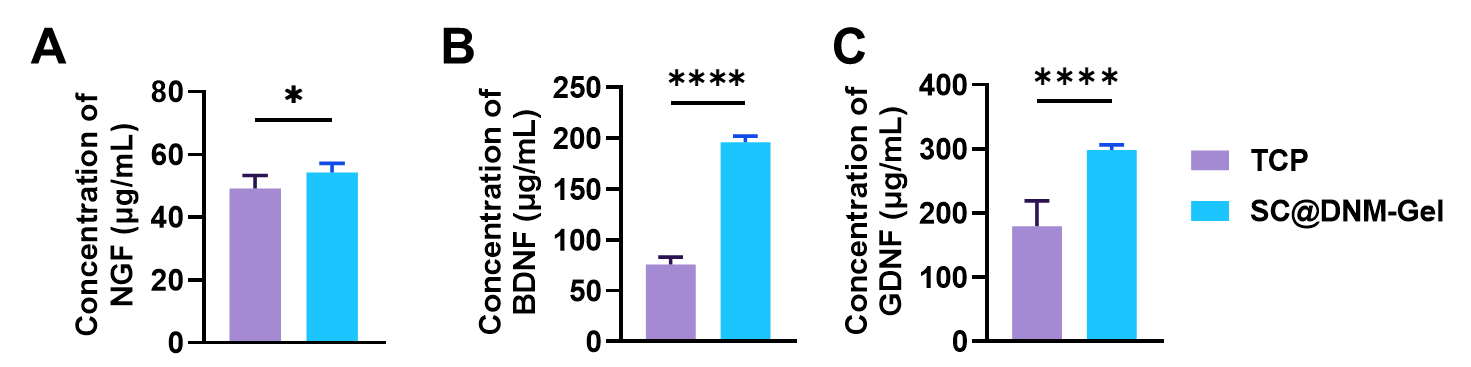


**Fig. S7.** Quantitative analysis of (A) NGF, (B) BDNF, and (C) GDNF concentrations in culture supernatant extracted from SCs cultured on TCP and in DNM-gel, respectively (n = 8).


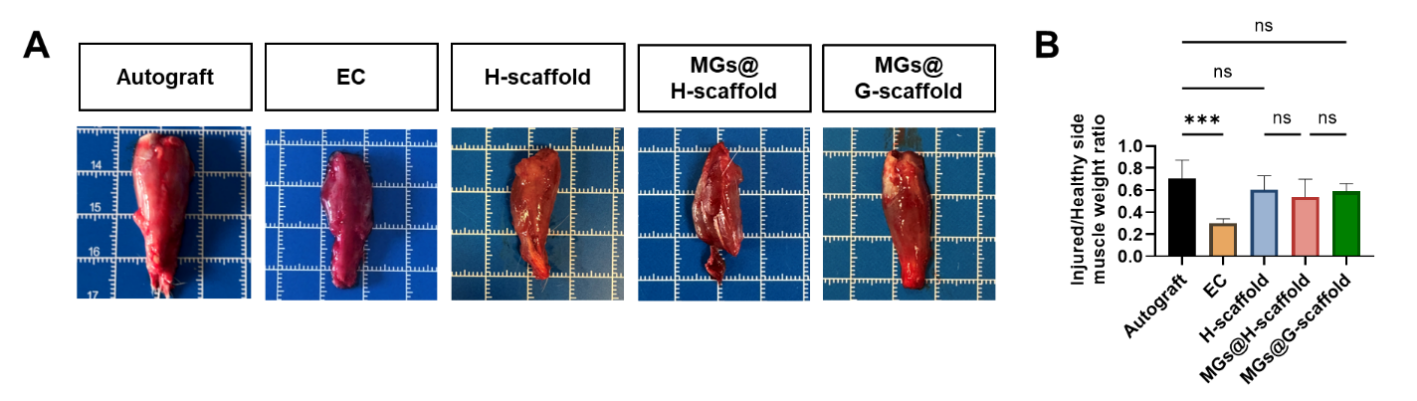


**Fig. S8.** (A) Representative photographs of the triceps surae muscles on the injured side with sciatic-nerve injury, harvested twelve weeks after surgery, and (B) Quantitative analysis of ratios by dividing the muscle weight of the injured side by that of the healthy side, n = 5, ****p* < 0.001, and ns represents not significant.
